# Comparative analyses and phylogenetic relationships of thirteen *Pholidota* species (Orchidaceae) inferred from complete chloroplast genomes

**DOI:** 10.1186/s12870-023-04233-8

**Published:** 2023-05-20

**Authors:** Lin Li, Wanyao Wang, Guoqiang Zhang, Kunlin Wu, Lin Fang, Mingzhi Li, Zhongjian Liu, Songjun Zeng

**Affiliations:** 1 Key Laboratory of South China Agricultural Plant Molecular Analysis and Genetic Improvement, Guangdong Provincial Key Laboratory of Applied Botany, South China Botanical Garden, Chinese Academy of Sciences, Guangzhou, 510650 China; 2 University of Chinese Academy of Sciences, Beijing, 100049 China; 3 Key Laboratory of National Forestry and Grassland Administration for Orchid Conservation and Utilization, The National Orchid Conservation Centre of China, Shenzhen, 518114 China; 4 Guangzhou Bio & Data Biotechnology Co., Ltd, Guangzhou, 510555 China; 5 Key Laboratory of National Forestry and Grassland Administration for Orchid Conservation and Utilization at College of Landscape Architecture, Fujian Agriculture, Fujian Agriculture and Forestry University, Fuzhou, 350002 China

**Keywords:** *Pholidota*, Chloroplast genome, Comparative analysis, Polyphyly, Phylogenetic relationship

## Abstract

**Background:**

The orchid genus *Pholidota* Lindl. ex Hook. is economically important as some species has long been used in traditional medicine. However, the systematic status of the genus and intergeneric relationships inferred from previous molecular studies are unclear due to insufficient sampling and lack of informative sites. So far, only limited genomic information has been available. The taxonomy of *Pholidota* remains unresolved and somewhat controversial. In this study, the complete chloroplast (cp.) genomes of thirteen *Pholidota* species were sequenced and analyzed to gain insight into the phylogeny of *Pholidota* and mutation patterns in their cp. genomes.

**Results:**

All examined thirteen *Pholidota* cp. genomes exhibited typical quadripartite circular structures, with the size ranging from 158,786 to 159,781 bp. The annotation contained a total of 135 genes in each cp. genome, i.e., 89 protein-coding genes, 38 tRNA genes, and eight rRNA genes. The codon usage analysis indicated the preference of A/U-ending codons. Repeat sequence analysis identified 444 tandem repeats, 322 palindromic repeats and 189 dispersed repeats. A total of 525 SSRs, 13,834 SNPs and 8,630 InDels were detected. Six mutational hotspots were identified as potential molecular markers. These molecular markers and highly variable regions are expected to facilitate future genetic and genomic studies. Our phylogenetic analyses confirmed the polyphyletic status of the genus *Pholidota*, with species grouped into four main clades: *Pholidota* s.s. was resolved as the sister to a clade containing species of *Coelogyne*; the other two clades clustered together with species of *Bulleyia* and *Panisea*, respectively; species *P. ventricosa* was placed at the basal position, deviated from all other species.

**Conclusion:**

This is the first study to comprehensively examine the genetic variations and systematically analyze the phylogeny and evolution of *Pholidota* based on plastid genomic data. These findings contribute to a better understanding of plastid genome evolution of *Pholidota* and provide new insights into the phylogeny of *Pholidota* and its closely related genera within the subtribe Coelogyninae. Our research has laid the foundation for future studies on the evolutionary mechanisms and classification of this economically and medicinally important genus.

**Supplementary Information:**

The online version contains supplementary material available at 10.1186/s12870-023-04233-8.

## Background

***Pholidota*** Lindl. ex Hook. is an orchid genus, which was described in 1825 by W.J. Hookers in his Exotic Flora. As current circumscribed, *Pholidota* belongs to the subtribe Coelogyninae, tribe Arethuseae of the subfamily Epidendroideae [1]. It consists of perennial herbaceous species and widely occurs in tropical and subtropical Asia from the Himalayas, southern China, through Southeast Asia, Malaysia, Philippines, Indonesia, to the western Pacific Islands, as well as northern Australia [1]. Currently, approximately 30 species have been recognized in the genus, with 15 species recorded from China [1–4].

*Pholidota* is of great economic and medicinal importance. Several species of this genus possess a broad range of medicinal properties and have been commonly used as folk herbal medicine for various medicinal purposes in China and India for many centuries [5–7]. There has been disagreement, however, regarding the taxonomy of this genus and its allies within Coelogyninae. The classification of genera in the Coelogyninae has mostly been based on a few easily observed characteristics. Saccate hypochile has been traditionally used as a diagnostic character to circumscribe *Pholidota* [1, 2]. Previous molecular data using partial sequences has shown that some traditionally circumscribed genera including *Coelogyne*, *Pholidota* and other related genera are not monophyletic and the relationships among them still remain elusive [8]. Likewise, despite *Panisea* has been usually recognized as a natural group, we find that some key characteristics for group identification such as basally sigmoid or shallowly saccate lip [1] are highly variable, with considerable overlap among the members of *Panisea* and *Pholidota.* The taxonomic complexity of these taxa is largely caused by high morphological diversity at both intrageneric and intergeneric levels and homoplasy of the major diagnostic characters. The taxonomic complexity of these groups makes generalizations difficult. Quite a few questions remain concerning these taxa including whether they should be considered distinct genera and how to circumscribe them.

Recent advances in molecular genomics and bioinformatics, particularly next-generation sequencing approaches present a phylogenomic framework for charting the diversity and evolution of angiosperm. Compared with the nuclear genome, the chloroplast genome has distinct features, e.g., maternal inheritance, high conservation and appropriate polymorphism. These properties make genetic polymorphism of the plastome a suitable source of molecular markers for a range of genetic and phylogenetic studies at different taxonomic levels in angiosperms [9–11]. It is increasingly realized that recent phylogenetic analyses using whole plastid genomes have largely deepen our understanding of the relationships in plant evolutionary history over the past three decades [12]. Despite its medicinal properties, genetic studies of *Pholidota* have been neglected. So far, information about *Pholidota* with regard to the genomic characteristics of the chloroplast genomes has been limited. The phylogenetic relationships between *Pholidota* and its closely related genera within Coelogyninae remain unclear. In the current study, we focused on (1) analyzing the thirteen cp. genome structural characteristics of *Pholidota*, (2) elucidating the genetic diversity and developing optimized markers for discriminating *Pholidota* species, (3) evaluating the phylogenetic position of *Pholidota* and discovering the most probable intra- and intergeneric relationships among *Pholidota* and its allies using chloroplast genome alignments.

## Results

### Characteristics of ***Pholidota*** plastomes

**Fig. 1:**
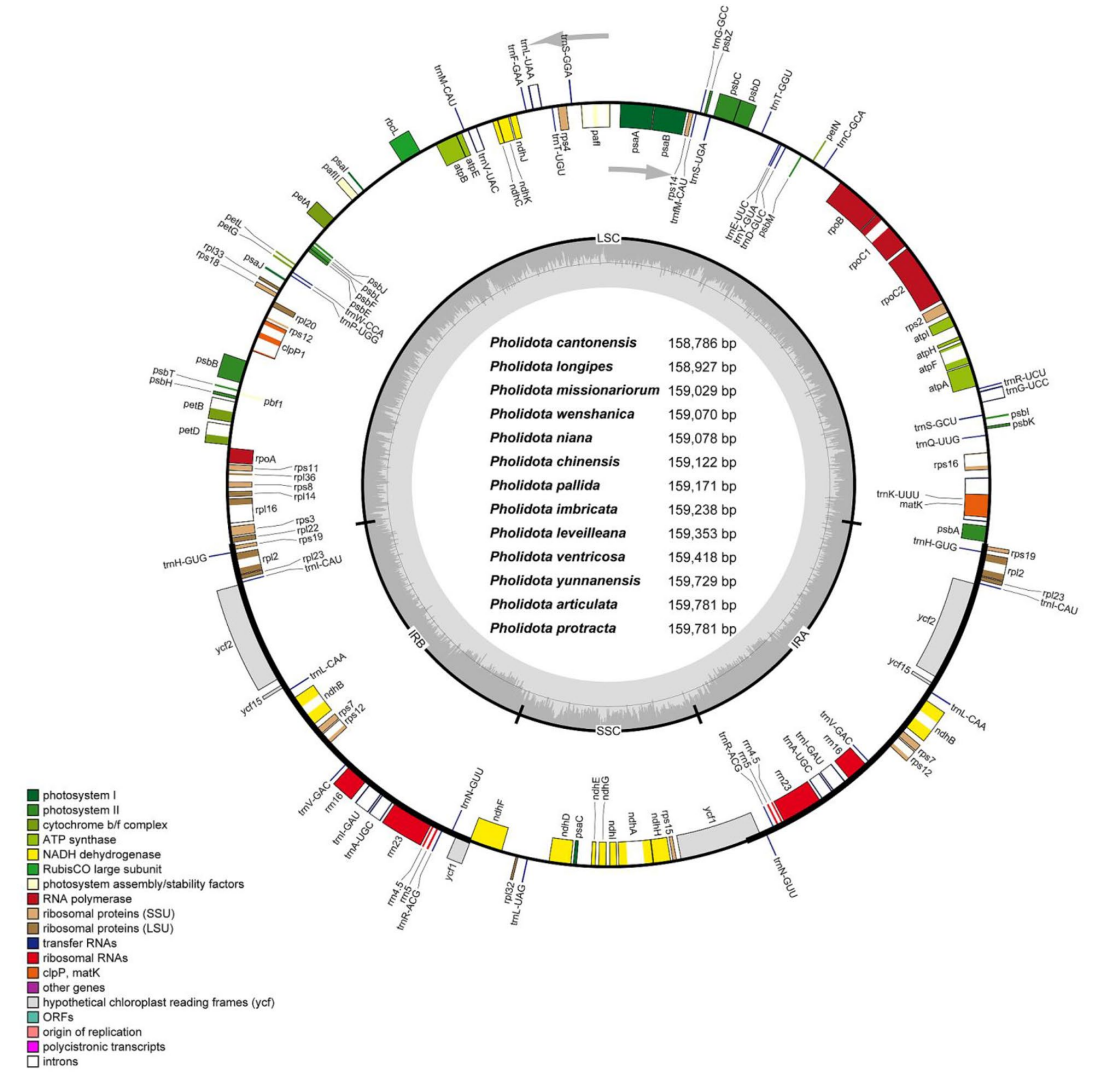
Circular gene map of the thirteen *Pholidota* chloroplast genomes. Genes shown inside of the circle are transcribed clockwise, while genes annotated outside the circle are transcribed counterclockwise. The innermost darker gray depicts the GC content. Genes belonging to different functional groups are labeled with different colors, as indicated in the legend

The graphical genome maps of the newly sequenced *Pholidota* cp genomes were provided in Fig. 1, generated using OGDRAW [13] and in Additional File 1: Fig. S1, using the GView server [14], respectively. All of the cp genomes exhibited a double-stranded circular quadripartite structure, comprising a large single copy region (LSC; 86,822 bp–87,756 bp), a small single copy region (SSC; 18,598 bp–18,851 bp), separated by a pair of inverted repeat regions (IRa and IRb; 26,470 bp–26,721 bp). The thirteen cp genomes ranged in size from 158,786 bp to 159,781 bp (Table 1). For each assembled cp genome, 135 genes were annotated, including 89 protein-coding genes, 38 tRNA genes and eight rRNA genes. The LSC region possessed 60 protein-coding and 21 tRNA genes, whereas, the SSC region only contained ten protein-coding and one tRNA genes. The overall GC content in these plastomes was similar, ranging from 37.27–37.47% and varied within the LSC, SSC and IR regions. The GC content in the IR regions (43.25–43.37%) was higher than those in the LSC (35.14–35.38%) and SSC (30.18–30.47%) regions (Fig. S1; Table 1). Within the IR regions, eight protein-coding genes (*rpl2*, *rpl23*, *rps7*, *rps12*, *rps19*, *ycf2*, *ycf15*, and *ndhB*), four rRNA genes (*rrn16*, *rrn23*, *rrn4.5* and *rrn5*), and eight tRNA genes (*trnA*-*UGC*, *trnH*-*GUG*, *trnI*-*CAU*, *trnI*-*GAU*, *trnL*-*CAA*, *trnN*-*GUU*, *trnR*-*ACG*, and *trnV*-*GAC*) were present in two copies. In all the plastomes of these species, *ycf1* gene was found to extend from IRa into the SSC region, and left a truncated copy at the junction of IRb/SSC. The *rps12* gene in *Pholidota* plastomes was arranged in a trans-spliced state, with 5’-end exon located in the LSC region and two 3’-end exons located in IR regions. Among all identified genes, eleven protein-coding genes (*atpF*, *ndhA*, two *ndhB*, *petB*, *petD*, *rpl2*, *rpl16*, *rpoC1*, *rps12* and *rps16*) and eight tRNA genes (two *trnA-UGC*, *trnG*-*UCC*, two *trnI*-*GAU*, *trnK*-*UUU*, *trnL*-*UAA* and *trnV*-*UAC*) each contained two exons, while the other four protein-coding genes (two *rps12*, *clpP1* and *paf1*) each contained three exons.

**Table 1:** Characteristics of chloroplast genomes of thirteen *Pholidota* species

Species	GenBank accession No.	Genome size (bp)	LSC		SSC		IR		GC (%)	CDS	tRNA	rRNA
			length (bp)	GC (%)	length (bp)	GC (%)	length (bp)	GC (%)				
*P*. *articulata*	ON880551	159,781	87,756	35.31	18,851	30.45	26,587	43.3	37.4	89	38	8
*P*. *cantonensis*	ON880552	158,786	86,996	35.38	18,762	30.47	26,514	43.37	37.47	89	38	8
*P*. *chinensis*	ON880553	159,122	86,905	35.34	18,809	30.34	26,704	43.27	37.41	89	38	8
*P*. *imbricata*	ON880554	159,238	87,454	35.32	18,806	30.32	26,489	43.31	37.39	89	38	8
*P*. *leveilleana*	ON880555	159,353	87,321	35.29	18,734	30.43	26,649	43.28	37.39	89	38	8
*P*. *longipes*	ON880556	158,927	86,822	35.25	18,791	30.18	26,657	43.25	37.34	89	38	8
*P*. *missionariorum*	ON880557	159,029	87,257	35.33	18,600	30.35	26,586	43.27	37.4	89	38	8
*P*. *niana*	ON880558	159,078	87,206	35.14	18,730	30.19	26,571	43.27	37.27	89	38	8
*P*. *pallida*	ON880559	159,171	87,399	35.27	18,832	30.34	26,470	43.33	37.37	89	38	8
*P*. *protracta*	ON880560	159,781	87,595	35.22	18,744	30.28	26,721	43.29	37.34	89	38	8
*P. ventricosa*	ON880561	159,418	87,408	35.17	18,598	30.42	26,706	43.29	37.34	89	38	8
*P. wenshanica*	ON880562	159,070	86,942	35.28	18,834	30.4	26,647	43.28	37.38	89	38	8
*P. yunnanensis*	ON880563	159,729	87,618	35.21	18,849	30.3	26,631	43.28	37.32	89	38	8

### Codon usage and amino acid frequencies

In order to investigate the codon usage pattern, the overall relative synonymous codon usage (RSCU) values of 13 *Pholidota* cp. genomes were calculated (summarized in Additional file 2: Figure S2 and Additional file 3: Table S1). Each cp. genome contained 64 codons with 61 sense codons encoding 21 amino acids (excluding three stop codons, UAA, UAG and UGA). The majority amino acids (19/21, 90.48%) were encoded by two to six synonymous codons, with the exception of two amino acids, methionine (Met) and tryptophan (Trp), which were encoded by a single codon (AUG and UGG, respectively). The most frequent amino acids encoded in the plastomes were arginine (Arg), leucine (Leu), and serine (Ser), which were each encoded by six synonymous codons. In contrast, Tryptophan (Trp) was the least common. The RSCU results showed that slightly more than half of the codons (30/59, 50.85%, excluding the start and stop codons) were more frequently used than expected (RSCU > 1). Almost all of the preferentially used codons (96.67%) ended with A/U except UUG, one of the codons for leucine (Leu). As expected, the use of the start codons AUG and UGG, encoding Met and Trp, exhibited no bias (RSCU = 1). The highest RSCU values (1.92–1.96) were exhibited in AGA encoding Arginine (Arg). The lowest RSCU value at approximately 0.33 was AGC encoding Serine (Ser).

### Examination of repeats and SSRs

**Fig. 2:**
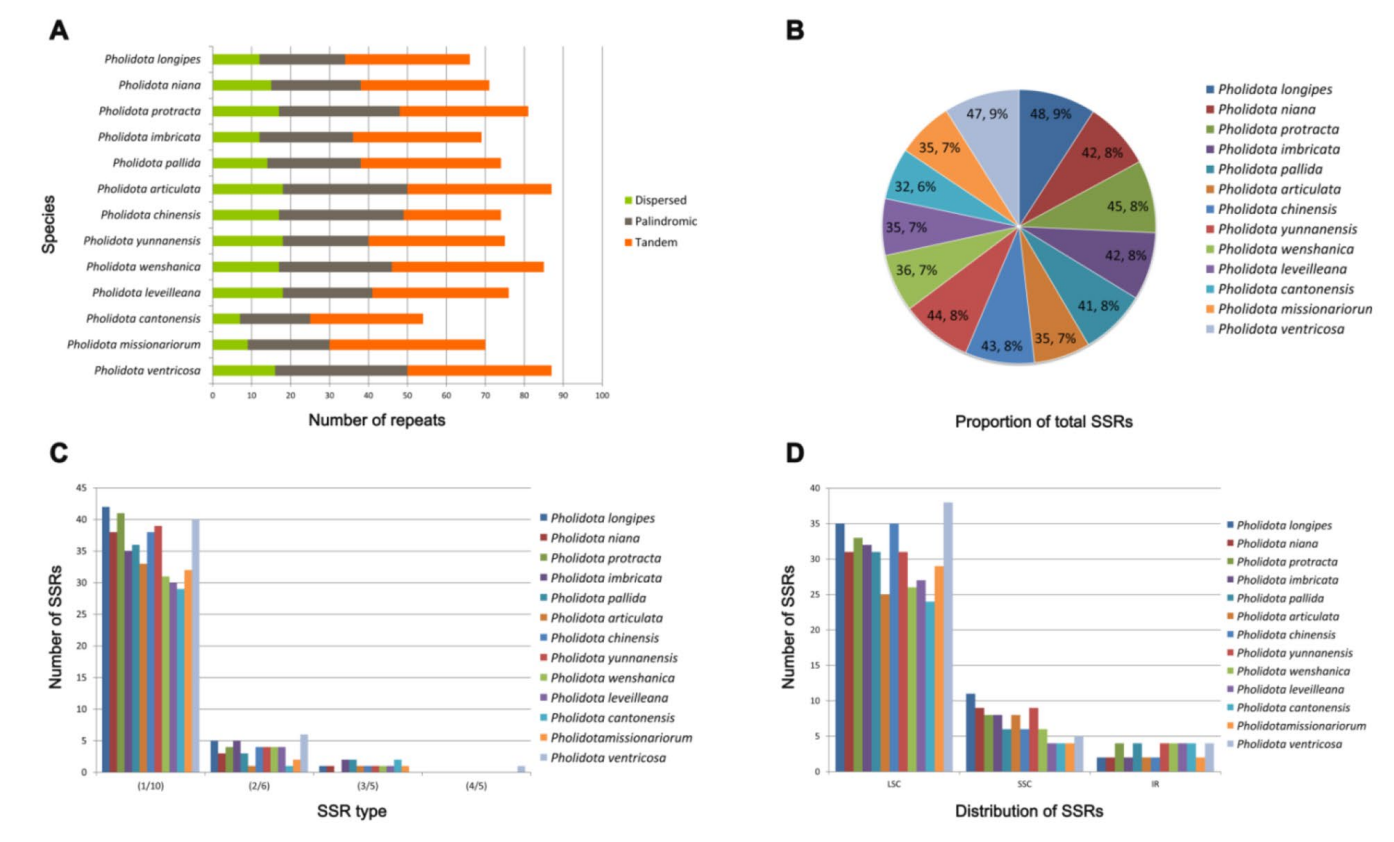
Repeat sequences and simple sequence repeats (SSRs) among the thirteen *Pholidota* cp. genomes. (**A**) Number of different types of repeats; (**B**) Distribution of repeats in each species; (**C**) Number of different types of SSRs; (**D**) Distribution of SSRs in different genome regions

Repetitive DNA sequences or repeats refer to homologous DNA fragments that occur as a multiple copy of nucleic acids in the genomes, which are the major components of eukaryotic genomes and considered to play an important role in genome stability and structural variation [15, 16]. In the current study, we employed REPuter and Tandem Repeats Finder to analyze the repetitive sequences in the thirteen cp. genomes of *Pholidota* species. In total, 955 repeats of at least 30 bp long per repeat unit were detected. We categorized these repeats into three types: tandem, dispersed and palindromic. Totally, our analysis identified 444 tandem repeats, 322 palindromic repeats and 189 dispersed repeats in the cp. genomes (Fig. 2A; Additional file 4: Table S2). The numbers of tandem repeats varied from 25 to 40; 17 to 33 for palindromic repeats and 7 to 18 for dispersed repeats. Generally, tandem repeats were found as the most prevalent type of repeats, with a proportion of 46.49%, followed by palindromic repeats (33.72%), whereas dispersed repeats were the least common and occupied the lowest portion of 19.79%. In particular, these repeats showed species-specific across the cp. genomes. The lengths of dispersed and palindromic repeats varied from 39 to 70 bp with the longest repeats presented in *P*. *protracta*. The maximum number (40) and minimum number (25) of tandem repeats were detected in *P*. *missionariorum* and *P*. *chinensis*, respectively. Meanwhile, *P*. *ventricosa* cp. genome had the highest frequency of palindromic repeats (33), whereas the lowest palindromic repeats (17) were detected in *P. cantonensis* cp. genome. Tandem repeats were found to be highly abundant and frequently dispersed in these genomes. As shown in Fig. 2A and Additional file 5: Table S3, more than half of the tandem repeats (59.46%) were localized in the LSC region, followed by IR regions (24.77%). The SSC region incorporated the least number of tandem repeats (15.77%) in the genome. Compared to the protein-coding regions (CDS, 23.2%), the intergenic spacer (IGS) regions harbored considerably more tandem repeats (67.57%).

Simple sequence repeats (SSRs), also known as microsatellites or short tandem repeats, refer to short tandemly repetitions of 1–6 base pairs. The SSR analysis of the *Pholidota* chloroplast genomes using the Perl script MISA detected 525 microsatellites in four main types, namely mono-, di-, tri- and tetra-nucleotide repeats. A comparison of total SSRs for each species is shown in Fig. 2B, with the numbers varied from 32 in *P*. *cantonensis* to 48 in *P*. *longipes* across the 13 cp. genomes (Additional file 6: Table S4). The most abundant SSRs were mono-nucleotides, accounting for approximately 88.38% of all SSRs. Only a small fraction consisted of di-nucleotide (8.76%) and tri-nucleotide (2.67%) repeat motifs. Almost all of the mono-nucleotides contained A /T repeat units (96.77%), with only 3.23% composed of C/G. Meanwhile, all of the di-nucleotides comprised only AT and TA motifs and deficient in CG. Notably, the distribution and frequency of different SSR motif types in these cp. genomes showed obvious differences. Tri-nucleotide repeats were not present in *P*. *protracta* and *P*. *ventricosa.* Only one tetra-nucleotide SSR (ATAG) was located in the cp. genome of *P*. *ventricosa* (Fig. 2C; Additional file 6: Table S4). In addition, the identified SSRs were found to be non-uniformly distributed in the chloroplast genomes of the genus *Pholidota*. The majority of SSRs resided in the LSC region (70.45–82.86%), followed by SSC region (10.64–22.92%), while a minority (4.17–12.5%) occurred within the IR regions (Fig. 2D; Additional file 6: Table S4).

### Mining of SNP and InDel markers

**Fig. 3:**
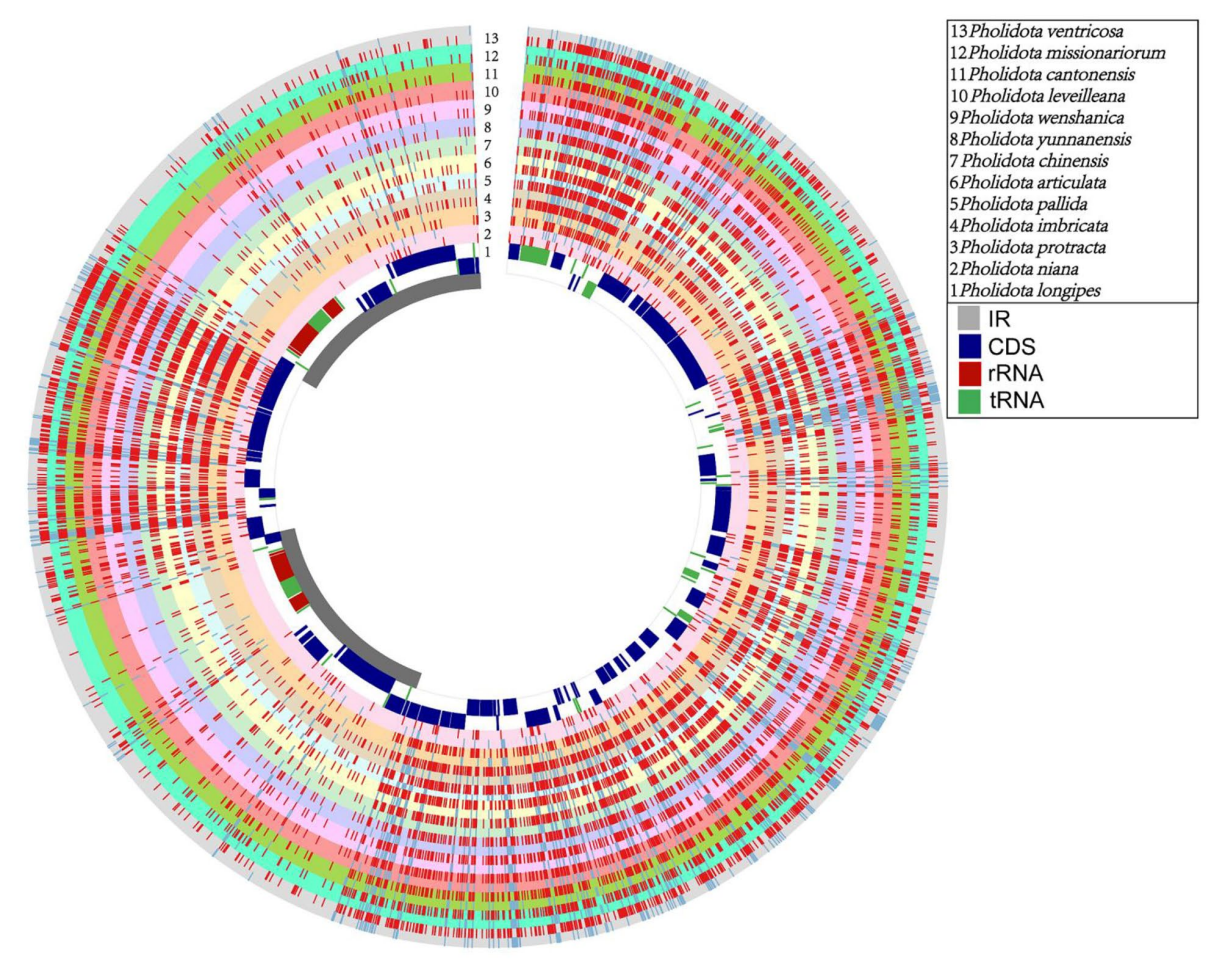
An overview of SNP and InDel variants among thirteen cp. genomes of *Pholidota* with P. longipes as a reference

Single-nucleotide polymorphisms (SNPs) and DNA insertions-deletions (InDels) are useful polymorphic markers for analysis of genetic diversity and genetic mapping. In this survey, we determined these genetic variants based on a comparison of 13 cp. genome alignments with the *P*. *longipes* cp. genome as a reference. In total, 22,464 mutations were identified, including 13,834 SNPs and 8,630 InDels (Additional file 7: Table S5). The number and distribution of SNPs and InDels detected in each species are displayed as bar graphs in different colors (Fig. 3, rings “2–13”). The number and frequency of SNPs and InDels varied considerably across the 13 *Pholidota* plastomes. The maximum number of SNPs was detected in the *P. ventricosa* cp. genome (1471) and the minimum number was found in the *P. niana* cp. genome (233). Similarly, the maximum number of InDels was also detected in *P. ventricosa* (916), and the lowest was observed in *P. niana* (264). The average numbers of SNPs and InDels were 1,064 and 267, respectively. Most of *Pholidota* species have 1,100 to 1,500 SNPs and 280 to 400 InDels. However, the SNPs in the cp. genomes of *P. niana* (233) and *P. protracta* (391) were significantly fewer than those in the other species. Similarly, fewer than 160 InDels were discovered in *P. niana* (62) and *P. protracta* (153) (Table S5). The low number of interspecific polymorphisms revealed high chloroplast sequence similarity among *P*. *longipes*, *P*. *niana* and *P*. *longipes*. Given the lower number of SNPs and InDels detected in *P*. *niana* and *P*. *protracta*, these species may be more closely related to each other.

The frequency and density of SNP/InDel loci identified in the genomic regions varied across the thirteen cp. genomes (Additional file 7: Table S5). A large number of variants resided in LSC regions, followed by the SSC region. The lowest number of polymorphic sites was noticed in the IR regions (Fig. 3). In addition, the overall distribution of SNPs and InDels among the cp. genomes shared a similar pattern. Sequences in the non-coding regions exhibited significantly higher divergence than that in the coding regions. For the non-coding regions, sequences of the intergenic spacer regions comprise a large majority of variants.

### Comparison of IR-SC border positions

**Fig. 4:**
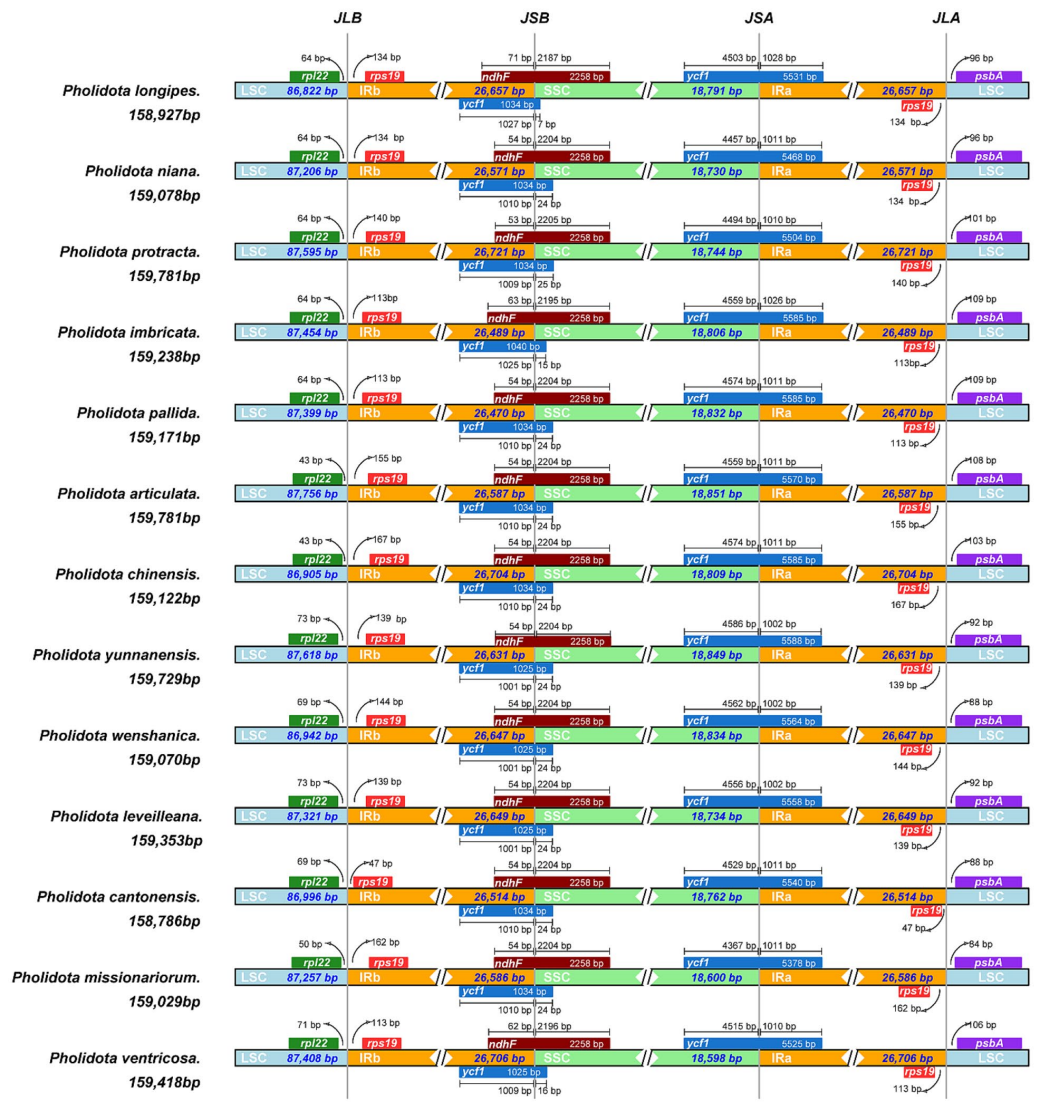
Comparison of the boundaries between the LSC, SSC and IR regions in the thirteen *Pholidota* cp. genomes. JLB: LSC/IRb junctions; JSB: SSC/IRb junctions; JSA: SSC/IRa junctions; JLA: LSC/IRa junctions. Genes are depicted by colored boxes. The numbers above or below the gene indicate the distance between the ends of the genes and the border sites

The border regions of LSC, SSC and IR regions of the thirteen *Pholidota* cp. genomes were compared to determine unique and common features (Fig. 4). In general, these cp. genomes exhibited relatively stable patterns with similar gene content and arrangement. The LSC/IRb boundary lied between *rpl22* and *rps19* genes, while the IRa/LSC border was located between *rps19* and *psbA* genes. Two intact copies of the *rps19* gene were present near the IR-LSC borders. In particular, the *ycf1* gene crossed the SSC/IRa boundary, leading to an incomplete duplication of this gene at the IRb/SSC border. In all cp. genomes sequenced, the partially duplicated *ycf1* gene spanned the IRb/SSC border and interlaced with the *ndhF* gene, extending to various lengths into the SSC region.

Although the length of the IR regions varied little across the thirteen *Pholidota* cp. genomes, ranging from 26,470 bp to 26,721 bp, the IR/SC boundary regions of these species present certain discrepancies. The SSC/IRa junction was situated in the *ycf1* coding region, with a size variation from 5,378 bp (*P*. *missionariorum*) to 5,588 bp (*P*. *yunnanensis*). At the SSC/IRa border, the *ycf1* gene extended into the SSC region, at varying lengths ranging from 4,367 bp in *P*. *missionariorum* to 4,568 bp in *P*. *yunnanensis*. The truncated copy of *ycf1* was largely located in the IRb region, with its one end extending into the SSC region, ranging from 7 bp (*P*. *longipes*) to 25 bp (*P*. *protracta*). In contrast, *ndhF* was mainly situated in the SSC region, partially overlapping with the duplicated *ycf1* gene. The *ndhF* gene showed the same length of 2,258 bp in all species, whereas the portion located in the IRb region varied in length from 53 bp in *P*. *protracta* to 71 bp in *P*. *longipes*. The distance between *rps19* and LSC/IRb border was 47 bp in *P. cantonensis*, whereas 167 bp in *P. chinensis*. The length from *rpl22* to the LSC/IRb border was 43 bp in *P. articulata* and *P. chinensis*, in contrast to 73 bp in *P. leveilleana* and *P. yunnanensis.* On the other side of the IRa/LSC boundary, *psbA* gene was found in the LSC region of all genomes but was located 84 bp (*P*. *missionariorum*) to 109 bp (*P*. *imbricata* and *P*. *pallida*) apart from the IRa/LSC border.

In general, the *Pholidota* cp. genomes showed obvious changes at the IR/SC boundaries and adjacent genes. Two genes *ycf1* and *ndhF* located at the IR/SSC junctions were found to be highly variable. The results also showed that some differences exist in the non-coding intergenic regions (*rpl22-rps19-psbA*).

### Genome sequence divergence among ***Pholidota*** species

**Fig. 5:**
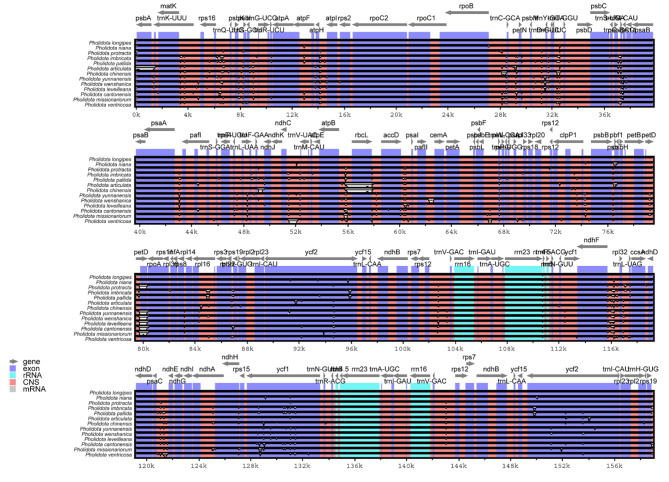
Comparative analysis of the thirteen *Pholidota* cp. genomes using the mVISTA program. The plastome of *P*. *longipes* was used as a reference. Grey arrows above the alignments indicate gene orientations. Genome regions are color-coded as protein-coding (exon; blue), ribosomal RNA (rRNA; cyan), and conserved non-coding sequences (CNS; pink)

The sequence divergence across the thirteen *Pholidota* cp. genomes were compared and plotted using mVISTA by aligning these cp. genomes with the annotated *P*. *longipes* cp. genome as a reference (Fig. 5). The whole-genome alignment revealed that sequence variations in the non-coding regions in orange bars were greater than that in the protein-coding regions (CDS) as colored in purple bars. The two IR regions were more conservative than the LSC and SSC regions. The highly divergent non-coding regions in these cp. genomes appeared in the intergenic spacer regions (IGS), such as *trnK*-*UUU*-*rps16*, *rps16-trnQ-UUG*, *trnR*-*UCU*-*atpA*, *atpF*-*atpH*, *trnC*-*GCA-petN*, *trnS*-*GGA*-*rps4*, *rps4*-*trnF*-*GAA*, *trnT-UGU*/*trnL-UAA*, *ndhC*-*trnV*-*UAC*, *atpB*-*rbcL*, *accD*-*psal*, *clpP1* intron, *psbB-psbT*, *petD*-*rpoA*, *rpl16*-*rps3*, *rps12*-*trnV*-*GAC*, *ndhF-rpl32*, *rpl32-trnL*-*UAG*, *psaC*-*ndhE*, and *trnV-GAC*. In CDS regions, some genes, such as *psbA, rps16*, *atpF*, *atpH*, *rbcL*, *psbT*, *petD*, *rpl16*, *rpl32*, *ycf1* and *ycf2* genes showed relatively high variations among these genomes. By contrast, all the rRNA genes were highly conserved when compared to the other genes.

**Fig. 6:**
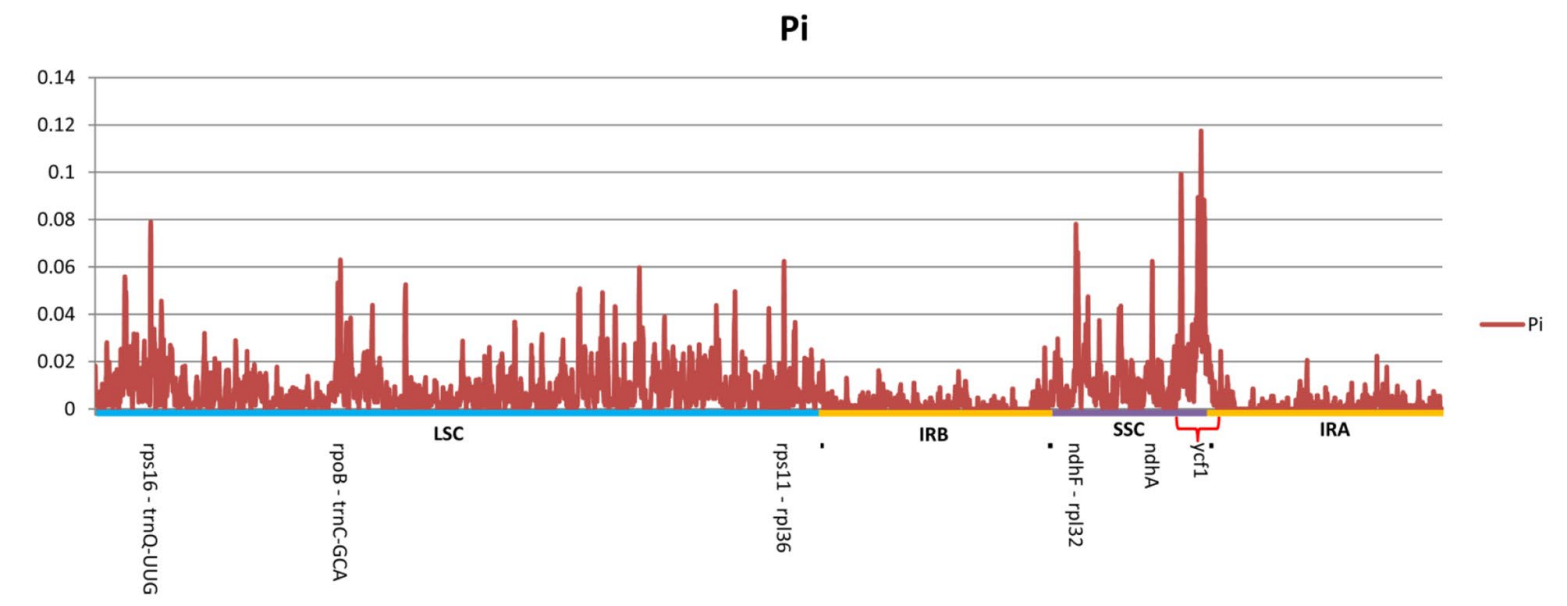
Nucleotide diversity (Pi) values across the thirteen *Pholidota* cp. genomes detected by sliding windows. The Y-axis shows the Pi values; the X-axis shows the genomic regions

To test divergence level within different regions of these cp. genomes, the nucleotide diversity (Pi) was measured by DnaSP within 600-bp windows (Fig. 6). The Pi values for each genome ranged from 0 to 0.1174. Of the protein-coding regions (CDS), the average Pi value was 0.0057. Compared to the coding regions, the intergenic spacer (IGS) regions showed comparably higher divergence levels, with an average Pi value of 0.0092. As expected, variations in the IR regions of the cp. genomes were considerably lower than that of SC regions. The SSC region showed the highest nucleotide diversity in view of its average Pi value of 0.0159, followed by LSC region, with an average of 0.0090, whereas the average Pi in the IR region was 0.0020.

We selected six highly variable regions in the cp. genomes with a nucleotide variability (Pi) higher than 0.06 (Fig. 6). These regions were four intergenic spacer regions: *rps16-trnQ-UUG* (0.0790), *ndhF*-*rpl32* (0.0758), *rpoB-trnC*-*GCA* (0.0630), *rps11-rpl36* (0.0624), and two plastid genes *ycf1* (0.1174), *ndhA* (0.0624) within the coding regions. Among these regions, *ndhF-rpl32* was located at the IRb/SSC junction, *ycf1* crossed the SSC/IRa border. *ndhA* was situated in the SSC region, whereas three of six (*rps16-trnQ-UUG, rpoB-trnC*-*GCA* and *rps11-rpl36*) were located in the LSC region (Fig. 6). These divergence hotspots could serve as significant genetic markers for species delimitation and phylogeographic analyses.

### Phylogenomic analysis

**Fig. 7:**
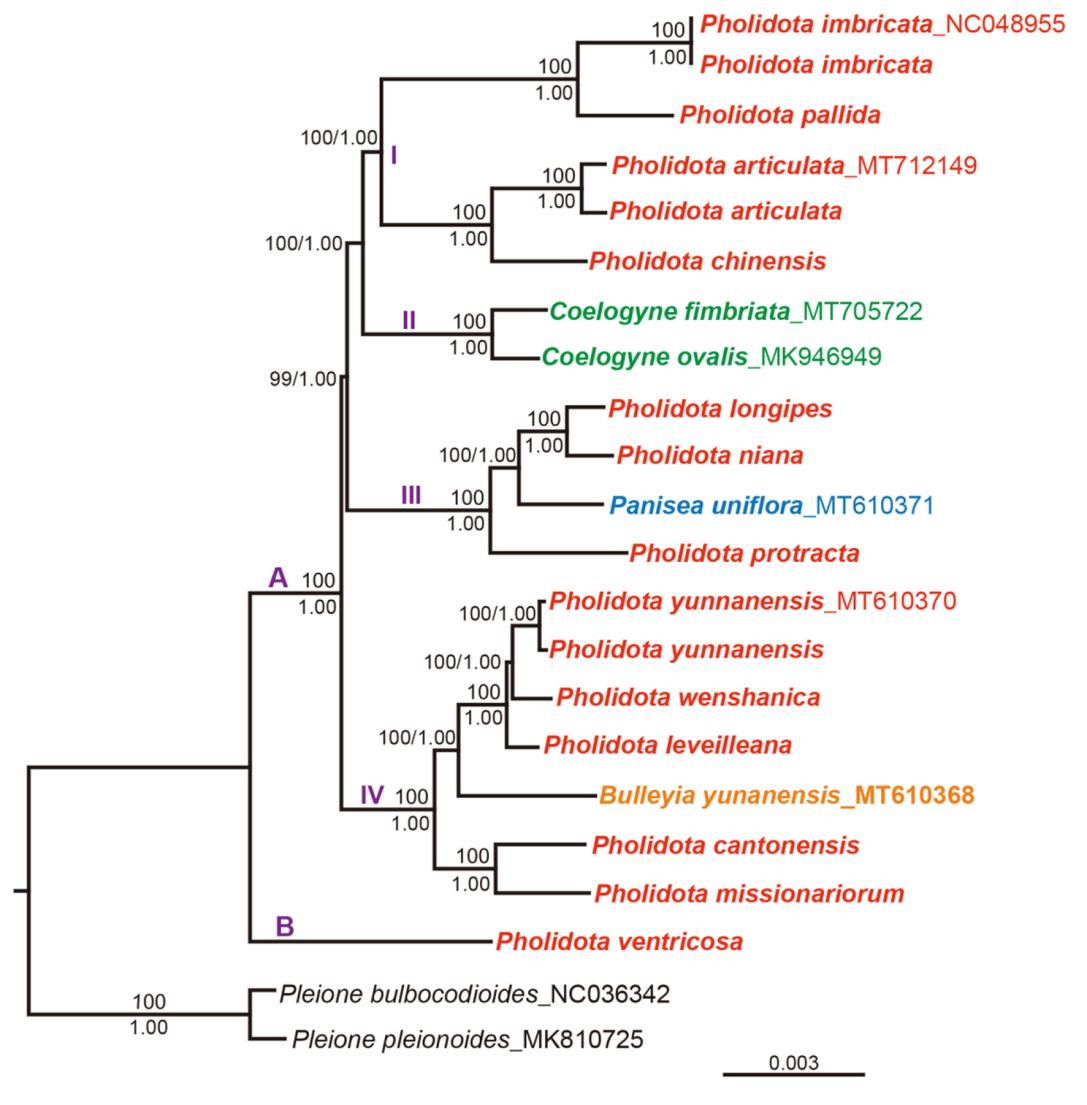
The ML phylogeny of *Pholidota* and its closely related genera in subtribe Coelogyninae inferred from 22 complete cp. genomes with *Pleione* species as the outgroups. Numbers (BS/PP) indicate bootstrap support values (BS) from maximum likelihood and posterior probabilities from Bayesian inference (PP). *Pholidota* taxa (including 13 species) are highlighted in red, *Coelogyne* species (including 2 species) are highlighted in green, *Panisea* species are highlighted in blue, and *Bulleyia* species are highlighted in orange

To determine the phylogenetic positions of *Pholidota* species and better clarify their evolutionary relationships, we constructed phylogenetic trees based on the complete plastome sequences of 22 species with maximum likelihood (ML) and Bayesian inference (BI) methods. In addition to the 13 newly sequenced genomes, three published cp. genomes of *Pholidota* and four published cp. genomes from other genera *Bulleyia*, *Coelogyne* and *Panisea* were also retrieved for analyses. Two species of genus *Pleione* were selected as outgroups based on previously reported relationships [17]. The ML and BI phylogenetic analyses yielded consistent topologies. The phylogram of the ML tree with the support values at the nodes is shown in Fig. 7.

The sampled species of *Pholidota* were generally separated into two main clades, namely clades A and B (Fig. 7). As the earliest differentiated lineage, clade B including an accession of species *P*. *ventricosa*, was placed as an unsupported sister to the remaining members of *Pholidota*. Clade A (PP = 1.0, BP = 100) containing most other sampled *Pholidota* species was further divided into three well-supported lineages, designated as subclades I, III, and IV that were found each related to the other genera of the subtribe Coelogyninae. Of the three subclades, subclade I consists of *P*. *imbricata* and *P*. *pallida*, forming the sister clades to *P*. *articulata*, and *P*. *chinensis*. Subclade II, which includes two sampled species of *Coelogyne* (*C*. *fimbriata*, *C*. *ovalis*), was strongly supported as the sister to subclade I (PP = 1.0, BP = 100). Within clade III, an accession of *Panisea uniflora*, fell within the lineage with accessions of *P. longipes*, *P. niana*, and *P. protracta*, constituting a strongly supported monophyletic group (PP = 1.0, BP = 100), with the former two as the closest sisters. Clade IV consisting of monotypic genus *Bulleyia*, with its sole representative, *B. yunnanensis*, together with six accessions of *Pholidota*, formed a robust monophyletic lineage (PP = 1.0, BP = 100). A small clade with accessions of *P. cantonensis* and *P. missionariorum* was placed as successive sisters to the clade with accessions of *P. leveilleana*, *P. wenshanica* and two accessions of *P. yunnanensis* (PP = 1.0, BP = 100).

## Discussion

### Structural features and plastome evolution of ***Pholidota***

All the newly assembled *Pholidota* plastomes presented the typical quadripartite structure of angiosperms and maintained a high degree of consistency among species (Fig. 1; Additional file 1: Fig. S1). Generally, no significant structural variations were detected with regard to the overall organization, gene composition and order. This is in accordance with the stability of plastome structure of most photosynthetic angiosperms [11]. The overall GC content of these plastomes had less fluctuation, ranging from 37.27 to 37.47%, and varied greatly among different genome regions. The IRs regions comprised the highest GC content (43.25–43.37%), whereas the SSC region had the lowest GC content (30.18–30.47%, Table 1). The GC content detected in the IR regions was consistently higher than the SC regions due to the presence of four copies of GC-rich rRNA genes (*rrn16*, *rrn23*, *rrn4.5*, *rrn5*) that aggregated in these regions. Interestingly, *P*. *cantonensis*, a small sized, cool to cold growing lithophytic or epiphytic species endemic to southern China, had a smallest chloroplast size (158,786 bp), but a highest GC content (37.47%). This phenomenon has been reported for other orchid species [17, 18]. Higher GC content is assumed to correlate with the increased tolerance to seasonally dry inhabitants or cold regions typical of a continental temperate climate for monocot species [19].

Codon usage bias (CUB) is a unique property of most genomes and varies greatly within and between organisms. It is expected to potentially influence genome evolution and widely used to understand the genetic and evolutionary patterns among different species [20, 21]. The results indicated that the overall codon usage patterns in the cp. genomes of thirteen *Pholidota* species were similar. The consistent utilization mode suggests the high conservation of *Pholidota* plastomes. In particular, these cp. genomes displayed a preference toward A/U-ending synonymous codons. This codon usage pattern was also found in other AT-rich angiosperms [22–24]. Furthermore, among 64 synonymous codons, AGA, encoding Arginine (Arg), GCU, encoding Alanine (Ala) and UUA, encoding (Leu) were found to be over-represented (RSCU > 1.8).

In general, the IR regions were relatively more conservative than the LSC and SSC regions and gene content of the IR borders among *Pholidota* plastomes was similar. However, slight variations of the border positions between the IR and SC regions were still detected (Fig. 4). For all species, *ycf1* gene crossed the IRa/SSC boundary regions, leading to an incomplete duplication or truncated copy (pseudogene) of this gene within IRs. In all these cp. genomes, the *ycf1* pseudogene overlapped with the *ndhF* gene in the IRb/SSC junction, creating various fragment lengths at the IRb region. Previous studies have shown that the stability of the IR/SC boundary regions is mainly correlated with the transformation of gene *ndhF* and/or *ycf1* [25–28]. We found that the IR/SC boundaries displayed minor fluctuations across *Pholidota* species. These changes were mainly associated with the different positions of *ndhF* and *ycf1*, together with the genes *rpl22*, *rps19* and *psbA* adjacent to LSC/IR and SSC/IR borders. The distribution and locations of gene types in these regions were highly consistent with previous studies for other orchid species of the subtribe Coelogyninae [17].

Despite their conservative structural organization, the cp. genomes of *Pholidota* varied in size from 158,786 bp (*P*. *cantonensis*) to 159,781 bp (*P*. *articulata* and *P*. *protracta*). The plastome size variation is usually attributed to the expansion and contraction of IR/SC boundary regions [10, 25, 29, 30]. The IR/SC boundary shifts will inevitably lead to the length variations of the four fundamental regions (LSC, SSC and two IRs), and in turn, creating overall plastome size variations. The size differences of *Pholidota* plastomes correspond to the IR/SC boundary shifts, which likely provide insights into their genome variation and evolutionary information. Various IR/SC boundary shifts are common evolutionary events and have a major impact on the plastome evolution of land plants [31]. It has been suggested that sequences of the two IR copies undergo frequent intramolecular recombination events to produce isomeric forms and maintain the gene complement of each SC region. The feature of IR regions may provide selective constraints on both sequence homogeneity and structural stability [29, 32, 33].

### Plastome polymorphisms and mutation hotspots of ***Pholidota***

Molecular markers based on DNA polymorphisms such as SSRs, SNPs and InDels are served as valuable genetic resources that extensively utilized for evaluation of genetic diversity and inferring molecular phylogeny [34, 35]. In this survey, a total of 525 SSRs were identified across the 13 *Pholidota* plastomes (Table S4). Mono-nucleotide SSRs were the most prevalent motifs and occupied the highest portion of all SSRs. Di- and tri-nucleotide SSRs were detected at a much lower frequency. Tri-nucleotide SSRs were present in eleven plastomes except for *P*. *protracta* and *P*. *ventricosa.* Tetra-nucleotide SSRs were found to be rare and species-specific, only detected in the cp. genome of *P*. *ventricosa.* In addition, most of the SSRs were composed of A/T repeat units, therefore it might contribute to the high AT richness of these cp. genomes. Polymorphism comparison of SNPs and InDels confirmed that these genetic markers are effective in identifying interspecific differentiation across these taxa. Most of variants were detected in *P*. *ventricosa*, whereas *P*. *niana* showed least variants (Fig. 3).

These molecular markers comprising SSRs, SNPs and InDels identified herein offer useful information into genetic variation, genomic evolution and species identification of *Pholidota*. Further examination indicated that these polymorphic variations were not evenly distributed in the cp. genomes. In comparison to the SSC and IR regions, the LSC region contained a higher amount of SSRs, SNPs and InDels (Figs. 2D and 3; Additional file 6: Table S4). Unsurprisingly, sequence variations were primarily located in non-coding regions such as intergenic spacer (IGS) region. Similar results have been shown in other chloroplast genomes of angiosperms [17, 30].

Nucleotide diversity (Pi) analysis of *Pholidota* plastomes showed that the LSC and SSC region exhibited comparably higher variations than the IR regions. For the CDS regions, the *ycf1* gene displayed the greatest level of divergence with the highest nucleotide diversity (Pi = 0.1174). Secondly, the intergenic regions (*rps16-trnQ-UUG*, *ndhF*-*rpl32*) with relatively high variability (> 0.07) were identified, with the former located in the LSC, and the latter located in the IRb/SSC border. Moreover, within the noncoding regions, highly divergent regions also included *rpoB-trnC*-*GCA* and *rps11-rpl36* (Fig. 6). Congruent with previous studies [17, 24, 28], Pi values were significantly higher for the *ndhF* and *ycf1* genes in coding regions. These hypervariable regions have potential values for future use as accurate and effective DNA barcoding markers to achieve authentication for traditional medicinal *Pholidota* plants.

### Phylogenetic relationships and implications

As proposed by De Vogel [2], the genus *Pholidota* was classified into nine sections based on morphological traits. Gravendeel et al. [8] proposed to include *Pholidota* in the “*Coelogyne s.s.* clade” and considered it to be closely related to the genus *Coelogyne*. The phylogeny of *Pholidota* has not been well studied and previous molecular studies based on a few molecular markers included only two samplings of *Pholidota* species. The phylogenetic placement of *Pholidota* and its phylogenetic relationship between the genus and other genera remain unclear. This study provides the first phylogeny of *Pholidota* inferred using the complete cp. genome data and proposes a robust phylogenetic hypothesis for 22 species representing five genera of the subtribe Coelogyninae.

Our phylogenomic analyses revealed that the genus *Pholidota* in traditional circumscription is highly polyphyletic, consisting of at least four clades (Fig. 7). *P*. *ventricosa* of *Pholidota* section *Chelonanthera* clearly formed an early-diverging lineage, isolated from the rest species sampled in the study. The species is easily distinguished from other sampled *Pholidota* members by having hairy pedicellate ovaries. The presence of hairs on the ovary is a uniquely derived character in Coelogyninae [8].

The remaining *Pholidota* species with glabrous ovaries fall into three major subclades (Fig. 7). Within subclade I, a small clade (*Pholidota* s.str. clade) comprising the generic type (*P*. *imbricata*) and representing the well-defined section *Pholidota* was well supported, and proved to cluster tightly together with its close relative *P*. *pallida.* The sister relationship between the *Pholidota* s.str. clade and a small clade composed by *P*. *articulata* (section *Articulatae*) and *P*. *chinensis* (section *Chinenses*) was also robustly recovered. As traditionally circumscribed, species of *Pholidota* are characterized by having a deeply saccate and boat-shaped lip hypochile [1, 2]. This characteristic usually makes it easily recognized from species of *Coelogyne* even for the amateur. Despite striking differences in their floral features, however, the *Pholidota s.str.* subclade appeared as paraphyletic relative to subclade II that includes two representatives of *Coelogyne* (*C*. *fimbriata* and *C*. *ovalis*). This result is in agreement with previous studies inferred from plastid fragments and ITS sequences where a similar relationship was weakly supported [8]. As the classifications proposed by previous studies based on morphological data are highly discordant with molecular data, a better understanding of their relationship would obviously require considerable additional sampling of *Coelogyne* members for further analyses.

The phylogenetic placement of *Panisea* had uncertain position in previous studies [8]. Here, we recovered strong support for a sister relationship between a representative of *Panisea*, *P. uniflora* and *P*. *protracta* of *Pholidota* section *Crinonia.* Besides, a small clade comprising *P*. *longipes* and *P*. *niana* was also clustered into the subclade III (Fig. 7). The grouping of these species is consistent with their morphological characteristics. In general, these species have similar floral bracts that are much shorter than pedicellate ovaries and persistent till long after anthesis. These results suggest that the distinctive bract characteristics are probably synapomorphies. Thus, we can infer that their nearest common ancestor possessed these features.

In particular, we investigated the systematic position of the monotypic genus *Bulleyia* using these cp. genomic sequences. Within the subclade IV (Fig. 7), *B. yunnanensis* was resolved as a sister to the clades comprising five *Pholidota* species of section *Chinenses* and section *Repentes*. Specifically, *P. cantonensis* and *P. missionariorum* of section *Repentes* formed a small clade that is sister to a larger clade made up of *B. yunnanensis*, *P. leveilleana*, *P. wenshanica* (sect. *Chinenses*) and two accessions of *P. yunnanensis* (sect. *Repentes*). These results indicate that neither section *Repentes* nor section *Chinenses* are monophyletic. Our data suggest a close relationship between *Bulleyia* and *Pholidota* species of these two sections. Species of *Bulleyia* is typically circumscribed based on a distinctive lip gradually narrowed at base into an incurved spur [1]. In fact, these characteristics associated with lip hypochile (e.g., saccate, sigmoid or spurred) that are traditionally used for the generic circumscription do not correspond to monophyletic groups. Considering that longer spur is usually presumed to represent an adaptation to pollinator [36], the individual floral characters of lip hypochile appear to vary across the taxa which are concerned with the pollination mechanism. These features might be evolutionarily labile and subject to frequent convergence and reversal. It should therefore be carefully reconsidered the morphological characters to circumscribe the clades identified by molecular data.

Our results highlight the homoplasious character of lip base that usually used for circumscribing generic boundaries. As these characters might represent homoplasies resulting from similar pollination systems rather than synapomorphies, the discriminatory characters must be chosen carefully in Coelogyninae. Owing to ambiguous diagnostic characters and similar floral features, the phylogenetic and taxonomic relationships of those species traditionally accommodated in *Pholidota* and its related genera are far more complex than previously expected. Thus, additional studies using more samples and more molecular data are needed to clarify the phylogeny and evolutionary history of *Pholidota* and its allies before a formal taxonomic revision.

## Conclusions

In this study, the complete chloroplast genomes of thirteen species of *Pholidota* were sequenced and assembled for the first time. These plastid genomes were found highly conservative in gene content, order and structure. Nonetheless, comparative analyses identified the divergence of the boundaries between LSC/SSC and IR regions. The multiple genetic markers, including repetitive sequences, SSRs, SNPs and InDels were proved to be highly efficient to evaluate the genetic diversity. Six hypervariable regions were selected that are potentially informative for barcoding, phylogenetic and population genetic researches. Our phylogenomic analyses yielded a well-resolved tree and identified highly supported novel phylogenetic relationships between *Pholidota* and its related genera. Our data provide strong support for the positions of *Bulleyia* and *Panisea.* Combined with the previous studies, we considered that the current taxonomy system of *Pholidota* needs to be improved and revised. The results further highlighted the advantage of plastome to infer phylogenies of putatively rapidly radiating groups. Moreover, the genomic data generated in the present study provide the basis for better understanding of the diversification of *Pholidota* and increasing the taxonomic resolution, species identification of *Pholidota*.

## Materials and methods

### Sampling, DNA isolation and sequencing

Fresh leaf materials were collected from healthy living plants cultivated at the greenhouses of the National Orchid Conservation & Research Center of Shenzhen (NOCC) and South China Botanical Garden, Chinese Academy of Science (SCBG, CAS). In total, 13 accessions of *Pholidota* representing major evolutionary lineages were sampled. Total genomic DNA was isolated from the young leaf using a modified CTAB method [37]. The purity and quality of DNA were detected by electrophoresis on the 1% Tris–acetate (TAE)–ethylenediamine tetraacetic acid (EDPA) agarose gel. Libraries were then constructed with the TruePrepTM DNA Library Prep Kit with insert sizes ranging from 250 to 350 bp according to the manufacturer’s protocol. These qualified libraries were sequenced on an Illumina HiSeq TM2500 platform by a 150 bp paired-end reads at the Novogene Bioinformatics Institute (Beijing, China). Approximately 10G raw data was generated for each sample.

### Plastome assembly and annotation

The raw sequencing reads were first filtered using FASTP [38] to remove reads containing ambiguous bases or low-quality bases. High-quality paired-end reads were then mapped to the reference genome sequences of Orchidaceae obtained from GenBank through Bowtie2 v.2.3.4.3 [39]. The gene coding sequence with maximum sequence coverage was utilized as a seed sequence for de-novo assembly by NOVOPlasty v4.2.1 [40] with the default parameters and manually adjusted to merge the overlapping reads into contigs. Gene annotation was initially performed using the online program DOGMA [41], GeSeq [42], tRNAscan [43] and then manually adjusted and confirmed using Geneious 9.1.8 [44] The circular chloroplast genome map was drawn using OGDRAW v.1.3.1 [13]. The complete plastome sequences of the 13 newly assembled *Pholidota* plastomes have been deposited in the NCBI GenBank database with the accession number ON880551–ON880563 (Table S1).

### Codon usage analyses

The relative synonymous codon usage (RSCU) of a codon represents the ratio of its observed frequency of utilization divided by the frequency expected if all the synonymous codons were used equally. An RSCU value equal to 1 denotes no bias in codon usage. RSCU value < 1.00 indicates a less-frequent usage, whereas RSCU value > 1.00 indicates a positive codon usage bias, as a codon is used more frequently than expected [45]. To determine the codon usage patterns of protein-coding sequences in the thirteen *Pholidota* plastomes, the RSCU values were calculated and obtained for each codon as previously described by Sharp and Li (1987) [45].

### Repeats and SSR analyses

REPuter v.2.74 program [46] (https://bibiserv.cebitec.uni-bielefeld.de/reputer/) was utilized to analyze dispersed (including forward, reverse, and complement repeat sequences) and palindrome repeats in *Pholidota* plastomes. For identification of these oligonucleotide repeats, the following conditions were used: a minimum repeat size of 30 bp; a hamming distance equal to 3 (i.e., 90% or greater sequence identity). In addition, Tandem Repeats Finder v.4.09 [47] (https://tandem.bu.edu/trf/trf.html) was employed to detect tandem repeats under default parameters. Simple sequence repeats (SSRs) or microsatellites in the genomes were examined using the Perl script-based program, MISA v.1.01 [48]. Different lengths of SSRs were determined by a settled minimum threshold of 10, 5, 4, 3, 3, and 3 repeat units for mono-, di-, tri-, tetra-, penta-, and hexa-nucleotides, respectively.

### Plastome comparison and sequence divergence analyses

BLAST Atlas on the GView server (https://server.gview.ca/) with 100 bp connection windows [14] was used for visualizing and assessing cp. genome features. For insertion-deletions (InDels) and single-nucleotide polymorphisms (SNPs) genetic markers, TBtools v.1.064 [49] was applied to create circos plot of cp. genomes. Expansion and contraction of IR regions of these cp. genomes was analysed and compared using the IRscope online program [50]. The divergent regions were plotted using Shuffle-LAGAN mode [51] included in mVISTA v.2.0 [52]. In order to further understand the sequence divergence, the nucleotide diversity (Pi) values of the 13 cp. genomes were calculated using DnaSP v6.12.03 software [53] with a sliding window analysis. The window length was set to 200 bp with a step size of 15 bp.

### Phylogenetic analyses

To better clarify the evolutionary relationships within the subtribe Coelogyninae, phylogenetic analyses were carried out based on 22 complete cp. genomes. Except for the 13 newly generated *Pholidota* cp. genomes in this study, seven published cp. genomes included two *Pholidota* species and five species of its closely related genera were downloaded from the NCBI database. *Pleione bulbocodioides* and *P*. *pleionoides* (Accession Nos. NC036342 and MK810725) were included as outgroups based on previous study [17]. All the genome sequences were aligned using MAFFT v7.313 [54] and adjusted manually by BioEdit [55]. Phylogenetic analyses were performed using both maximum likelihood (ML) and Bayesian inference (BI) methods. The maximum likelihood (ML) tree was generated using IQ-TREE version 1.6.12 [56] and web server (http://iqtree.cibiv.univie.ac.at). The best-fitting nucleotide substitution model TVM + F + R3 was determined using the Akaike Information Criterion (AIC) by ModelFinder [57] in the IQ-TREE package and 1,000 bootstrap replicates. Meanwhile, Bayesian inference tree was produced using MrBayes v.3.2.7 [58], based on Markov Chain Monte Carlo (MCMC) runs for 1,000,000 generations, employing the TVM + F + R3 model of nucleotide substitution, as determined by ModelTest-NG 0.1.6 [59]. These trees were sampled every 1,000 generations with the first 25% sampled trees discarded in the burn-in period.

## Electronic supplementary material

Below is the link to the electronic supplementary material.

**Additional file 1: Figure S1.**?A graphical circular map generated using the Gview server, showing a full view of the thirteen *Pholidota plastomes* with *P. longipes* as a reference. The innermost ring shows the genome size in kbp, followed by GC skew in purple, GC content in black, protein-coding genes on both the forward and reverse strand. The remaining rings display BLAST comparisons of plastome sequences. From the inside to the outside: *P. ventricosa, P. missionariorum, P. cantonensis, P. leveilleana, P. wenshanica, P. yunnanensis, P. chinensis, P. articulata, P. pallida, P. imbricata, P. protracta, P. niana, P. longipes*. The similar and divergent locations are shown in continuous and interrupted track lines, respectively. The lightly screened areas from the inside radically out denote divergent regions with high levels of variations.

**Additional file 2: Figure S2.** Codon usage frequency based on relative synonymous codon usage (RSCU) values in the thirteen *Pholidota* cp genomes.

**Additional file 3: Table S1.** Codon frequencies and relative synonymous codon usage (RSCU) values of the thirteen *Pholidota* cp genomes.

**Additional file 4: Table S2.** Types and numbers of long repeats in the thirteen *Pholidota* cp genomes.

**Additional file 5: Table S3.** Distribution of tandem repeats in the thirteen *Pholidota* cp genomes.

**Additional file 6: Table S4.** Types and numbers of SSRs distributed in LSC, SSC, IR regions based on nucleotide repeat units in the thirteen *Pholidota* plastomes.

**Additional file 7: Table S5.** The statistics of SNPs and InDels identified for each plastome alignment with *Pholidota* cp genome as a reference.

## Data Availability

The datasets generated in this study have been deposited in the NCBI database (https://www.ncbi.nlm.nih.gov/genbank/) with GenBank accession numbers shown in Table 1 (ON880551-ON880563). All data generated or analysed during this study are included in this published article and the supplementary information files.

## Abbreviations

AIC: Akaike information criterion
BI: Bayesian Inference
BLAST: Basic local alignment search tool
CDS: Protein-coding sequences
cp: Chloroplast
CTAB: Cetyl trimethylammonium bromide
DnaSP: DNA sequences polymorphism
GC: Guanine-cytosine
IGS: Intergenic sequences
Indel: Insertions/deletions
IR: Inverted repeat
IRA: Inverted repeat A
IRB: Inverted repeat B
IRs: Inverted repeat regions
ITS: Internal transcribed spacer of ribosomal DNA
LSC: Large single copy
ML: Maximum Likelihood
NCBI: National Center for Biotechnology Information
OGDRAW: OrganellarGenomeDRAW
Pi: Nucleotide diversity
rRNAs: Ribosomal RNAs
RSCU: Relative synonymous codon usage
SNP: Single nucleotide polymorphism
SSC: Small single copy
SSRs: Simple sequence repeats
tRNAs: Transfer RNAs.

## References

[1] Pridgeon AM, Cribb PJ, Chase MW, Rasmussen FN (2005). Genera Orchidacearum, Epidendroideae (part one).

[2] De Vogel EF (1988). Revisions in Coelogyninae (Orchidaceae) III. The genus *Pholidota*. Orchid Monogr.

[3] Chen SC, Wood JJ, Wu ZY, Raven PH, Hong DY (2009). *Pholidota* Lindl. Ex hook. Flora of China.

[4] Li L, Qin M, Wang WY, Zeng SJ, Zhang GQ, Liu ZJ. The taxonomic identities of Pholidota wenshanica and P. subcalceata (Orchidaceae, Coelogyninae). Phytokeys 2019;136:97–106. 10.3897/phytokeys.136.46705 PMC6933024 31892815

[5] Wang J, Matuszaki K, Kitanaka S (2006). Stilbene derivatives from *Pholidota chinensis* and their anti-inflammatory activity. Chem Pharm Bull.

[6] Guo XY, Wang J, Wang NL, Kitanaka S, Yao XS (2007). 9, 10- dihydrophenanthrene derivatives from *Pholidota yunnanensis* and scavenging activity on DPPH free radical. J Asian Natural Prod Res.

[7] Sharma C, Irshad S, Khatoon S, Arya KR (2017). Pharmacognostical evaluation of indian folk-traditional plants *Coelogyne cristata* and *Pholidota articulata* used for healing fractures. Indian J Exp Biol.

[8] Gravendeel B, Chase MW, De Vogel EF, Roos M, Mes THM, Bachmann K (2001). Molecular phylogeny of *Coleogyne* (Epidendroideae; Orchidaceae) based on plastid RFLPs, matK, and nuclear ribosomal ITS sequences: evidence for polyphyly. Am J Bot.

[9] Ravi V, Khurana JP, Tyagi AK, Khurana P (2008). An update on chloroplast genomes. Plant Syst Evol.

[10] Yang JB, Tang M, Li HT, Zhang ZR, Li DZ (2013). Complete chloroplast genome of the genus *Cymbidium*: lights into the species identification, phylogenetic implications and population genetic analyses. BMC Evol Biol.

[11] Daniell H, Lin CS, Yu M, Chang WJ (2016). Chloroplast genomes: diversity, evolution, and applications in genetic engineering. Genome Biol.

[12] Gitzendanner MA, Soltis PS, Yi TS, Li DZ, Soltis DE, Chaw SM, Jansen RK (2018). Plastome phylogenetics: 30 years of inferences into plant evolution. Advances in botanical research.

[13] Greiner S, Lehwark P, Bock R (2019). OrganellarGenomeDRAW (OGDRAW) version 1.3.1: expanded toolkit for the graphical visualization of organellar genomes. Nucleic Acids Res.

[14] Petkau A, Stuart-Edwards M, Stothard P, van Domselaar G (2010). Interactive microbial genome visualization with GView. Bioinformatics.

[15] Milligan BG, Hampton JN, Palmer JD (1989). Dispersed repeats and structural reorganization in subclover chloroplast DNA. Mol Biol Evol.

[16] Mehrotra S, Goyal V (2014). Repetitive sequences in plant nuclear DNA: types, distribution, evolution and function. Genom Proteom Bioinf.

[17] Li L, Wu QP, Fang L, Wu KL, Li MZ, Zeng SJ (2022). Comparative chloroplast genomics and phylogenetic analysis of *Thuniopsis* and closely related genera within Coelogyninae (Orchidaceae). Front Genet.

[18] Trávníček P, Čertner M, Ponert J, Chumová Z, Jersáková J, Suda J (2019). Diversity in genome size and GC content shows adaptive potential in orchids and is closely linked to partial endoreplication, plant life-history traits and climatic conditions. New Phytol.

[19] Šmarda P, Bureš P, Horová L, Leitch IJ, Mucina L, Pacini E, Tichý L, Grulich V, Rotreklová O (2014). Ecological and evolutionary significance of genomic GC content diversity in monocots. Proc Natl Acad Sci USA.

[20] Morton BR (2003). The role of context-dependent mutations in generating compositional and codon usage bias in grass chloroplast DNA. J Mol Evol.

[21] Batzman M, Margalit H (2011). Variation in global codon usage bias among prokaryotic organisms is associated with their lifestyles. Genome Biol.

[22] Eguiluz M, Rodrigues NF, Guzman F, Yuyama P, Margis R (2017). The chloroplast genome sequence from Eugenia uniflora, a Myrtaceae from Neotropics. Plant Syst Evol.

[23] Majeed A, Kaur H, Bhardwaj P (2020). Selection constraints determine preference for A/U-ending codons in Taxus contorta. Genome.

[24] Zheng G, Wei L, Ma L, Wu Z, Gu C, Chen K (2020). Comparative analyses of chloroplast genomes from 13 *Lagerstroemia* (Lythraceae) species: identification of highly divergent regions and inference of phylogenetic relationships. Plant Mol Biol.

[25] Kim KJ, Lee HL (2004). Complete chloroplast genome sequences from korean ginseng (*Panax schinseng Nees*) and comparative analysis of sequence evolution among 17 vascular plants. DNA Res.

[26] Luo J, Hou BW, Niu ZT, Liu W, Xue QY, Ding XY (2014). Comparative chloroplast genomes of photosynthetic orchids: insights into evolution of the Orchidaceae and development of molecular markers for phylogenetic applications. PLoS ONE.

[27] Kim HT, Kim JS, Moore MJ, Neubig KM, Williams NH, Whitten WM, Kim JH (2015). Seven new complete plastome sequences reveal rampant independent loss of the ndh gene family across orchids and associated instability of the inverted repeat/small single-copy region boundaries. PLoS ONE.

[28] Dong WL, Wang RN, Zhang NY, Fan WB, Fang MF, Li ZH (2018). Molecular evolution of chloroplast genomes of orchid species: insights into phylogenetic relationship and adaptive evolution. Int J Mol Sci.

[29] Yi DK, Kim KJ (2012). Complete chloroplast genome sequences of important oilseed crop *Sesamum indicum* L. PLoS ONE.

[30] Li R, Ma PF, Wen J, Yi TS (2013). Complete sequencing of five Araliaceae chloroplast genomes and the phylogenetic implications. PLoS ONE.

[31] Zhu A, Guo W, Gupta S, Fan W, Mower JP (2016). Evolutionary dynamics of the plastid inverted repeat: the effects of expansion, contraction, and loss on substitution rates. New Phytol.

[32] Palmer JD, Bogorad L, Vasil IK (1991). Plastid chromosomes: structure and evolution.

[33] Goulding SE, Olmstead RG, Morden CW, Wolfe KH (1996). Ebb and flow of the chloroplast inverted repeat. Mol Gen Genet.

[34] Ellegren H (2004). Microsatellites: simple sequences with complex evolution. Nat Rev Genet.

[35] Doorduin L, BGravendeel, Lammers Y, Ariyurek Y, Chin-A-Woeng T, Vrieling K (2011). The complete chloroplast genome of 17 individuals of pest species *Jacobaea vulgaris*: SNPs, microsatellites and barcoding markers for population and phylogenetic studies. DNA Res.

[36] Van der Niet T, Johnson SD (2012). Phylogenetic evidence for pollinator-driven diversification of angiosperms. Trends Ecol Evol.

[37] Doyle JJ (1987). A rapid DNA isolation procedure for small quantities of fresh leaf tissue. Phytochem Bull.

[38] Chen S, Zhou Y, Chen Y, Gu J (2018). Fastp: an ultra-fast all-in-one FASTQ preprocessor. Bioinformatics.

[39] Langmead B, Salzber SL (2012). Fast gapped-read alignment with Bowtie 2. Nat Methods.

[40] Dierckxsens N, Mardulyn P, Smits G (2017). NOVOPlasty: de novo assembly of organelle genomes from whole genome data. Nucleic Acids Res.

[41] Wyman SK, Jansen RK, Boore JL (2004). Automatic annotation of organellar genomes with DOGMA. Bioinformatics.

[42] Tillich M, Lehwark P, Pellizzer T, Ulbricht-Jones ES, Fischer A, Bock R, Stephan G (2017). GeSeq - versatile and accurate annotation of organelle genomes. Nucleic Acids Res.

[43] Chan PP, Lowe TM (2019). tRNAscan-SE: searching for tRNA genes in genomic sequences. Methods Mol Biol.

[44] Kearse M, Moir R, Wilson A, Stones-Havas S, Cheung M, Sturrock S (2012). Geneious basic: an integrated and extendable desktop software platform for the organization and analysis of sequence data. Bioinformatics.

[45] Sharp PM, Li WH (1987). The codon adaptation indexa measure of directional synonymous codon usage bias, and its potential applications. Nucleic Acids Res.

[46] Kurtz S, Choudhuri JV, Ohlebusch E, Schleiermacher C, Stoye J, Giegerich R (2001). REPuter: the manifold applications of repeat analysis on a genomic scale. Nucleic Acids Res.

[47] Benson G (1999). Tandem repeats finder: a program to analyze DNA sequences. Nucleic Acids Res.

[48] Beier S, Thiel T, Münch T, Scholz U, Mascher M (2017). MISA-web: a web server for microsatellite prediction. Bioinformatics.

[49] Chen CJ, Chen H, Zhang Y, Thomas HR, Frank MH, He YY, Xia R (2020). TBtools: an integrative toolkit developed for interactive analyses of big biological data. Mol Plant.

[50] Amiryousefi A, Hyvönen J, Poczai P (2018). IRscope: an online program to visualize the junction sites of chloroplast genomes. Bioinformatics.

[51] Brudno M, Do CB, Cooper GM, Kim MF, Davydov E, Green ED, Sidow A, Batzoglou S, Program NCS (2003). LAGAN and Multi-LAGAN: efficient tools for large-scale multiple alignment of genomic DNA. Genome Res.

[52] Frazer KA, Pachter L, Poliakov A, Rubin EM, Dubchak I (2004). VISTA: computational tools for comparative genomics. Nucleic Acids Res.

[53] Rozas J, Ferrer-Mata A, Sánchez-DelBarrio JC, Guirao-Rico S, Librado P, Ramos-Onsins SE, Sánchez-Gracia A (2017). DnaSP 6: DNA sequence polymorphism analysis of large data sets. Mol Biol Evol.

[54] Katoh K, Standley DM (2013). MAFFT multiple sequence alignment software version 7: improvements in performance and usability. Mol Biol Evol.

[55] Hall TA, BioEdit. A user-friendly biological sequence alignment editor and analysis program for Windows 95/98/NT. Nucleic Acids Symp. Ser. 1999;41:95–8.

[56] Nguyen LT, Schmidt HA, Von Haeseler A, Minh BQ (2015). IQ-TREE: a fast and effective stochastic algorithm for estimating maximum-likelihood phylogenies. Mol Biol Evol.

[57] Kalyaanamoorthy S, Minh BQ, Wong TKF, Von Haeseler A, Jermiin LS (2017). ModelFinder: fast model selection for accurate phylogenetic estimates. Nat Methods.

[58] Ronquist F, Teslenko M, Van Der Mark P, Ayres DL, Darling A, Höhna S, Larget B, Liu L, Suchard MA, Huelsenbeck JP (2012). MrBayes 3.2: efficient bayesian phylogenetic inference and model choice across a large model space. Syst Biol.

[59] Darriba D, Posada D, Kozlov AM, Stamatakis A, Morel B, Flouri T (2019). ModelTest-NG: a New and Scalable Tool for the selection of DNA and protein evolutionary models. Mol Biol Evol.

