# Supplementary figures and images for: Comparative analyses and phylogenetic relationships of thirteen Pholidota species (Orchidaceae) inferred from complete chloroplast genomes
Source: BMC Plant Biol. 2023 May 20;23:269. doi: 10.1186/s12870-023-04233-8 (PMC10199590; doi:10.1186/s12870-023-04233-8)

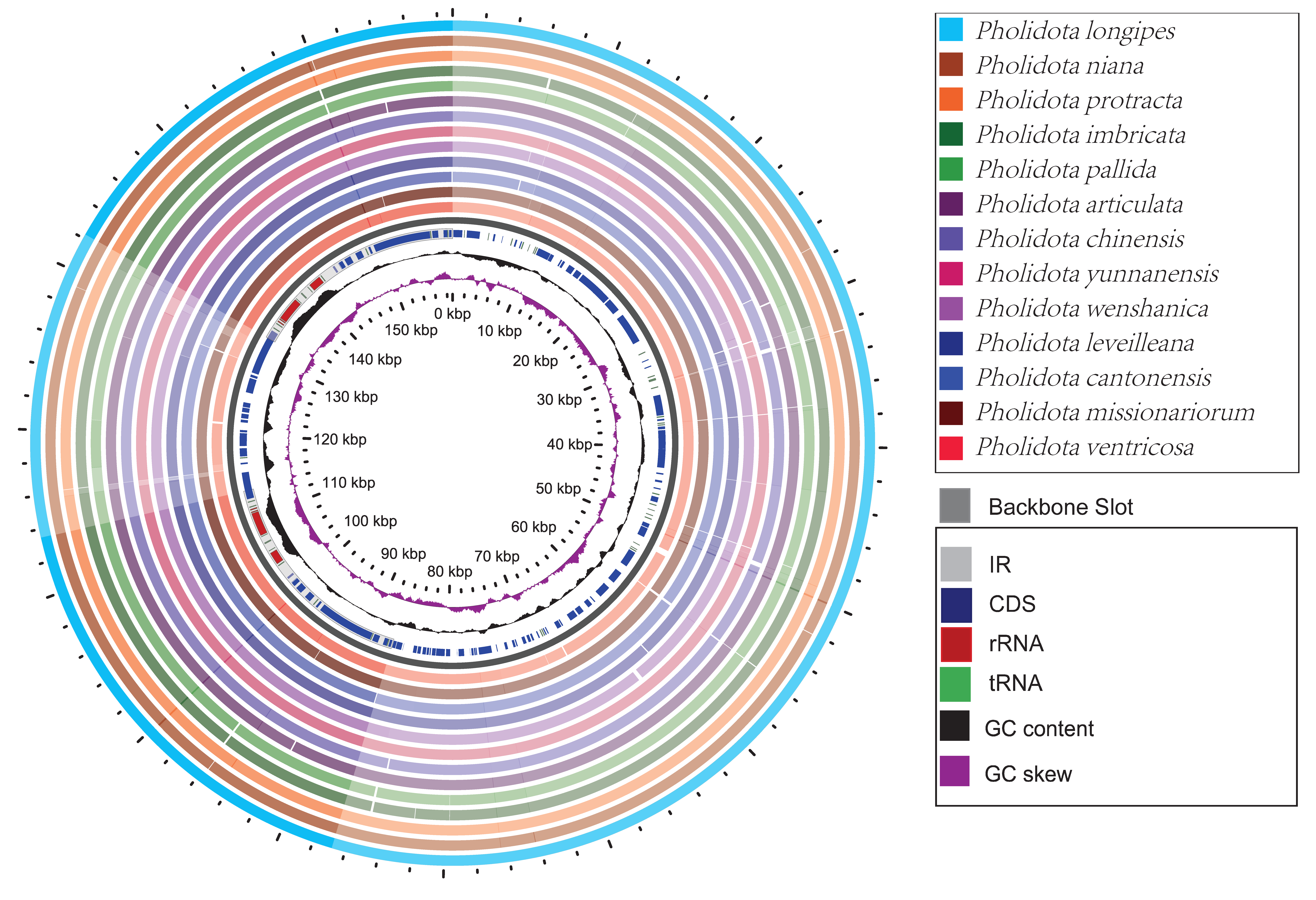

Supplement: Supplementary file 1 — Additional file 1: Figure S1.?A graphical circular map generated using the Gview server, showing a full view of the thirteen Pholidota plastomes with P. longipes as a reference. The innermost ring shows the genome size in kbp, followed by GC skew in purple, GC content in black, protein-coding genes on both the forward and reverse strand. The remaining rings display BLAST comparisons of plastome sequences. From the inside to the outside: P. ventricosa, P. missionariorum, P. cantonensis, P. leveilleana, P. wenshanica, P. yunnanensis, P. chinensis, P. articulata, P. pallida, P. imbricata, P. protracta, P. niana, P. longipes. The similar and divergent locations are shown in continuous and interrupted track lines, respectively. The lightly screened areas from the inside radically out denote divergent regions with high levels of variations. [file 12870_2023_4233_MOESM1_ESM.tif]

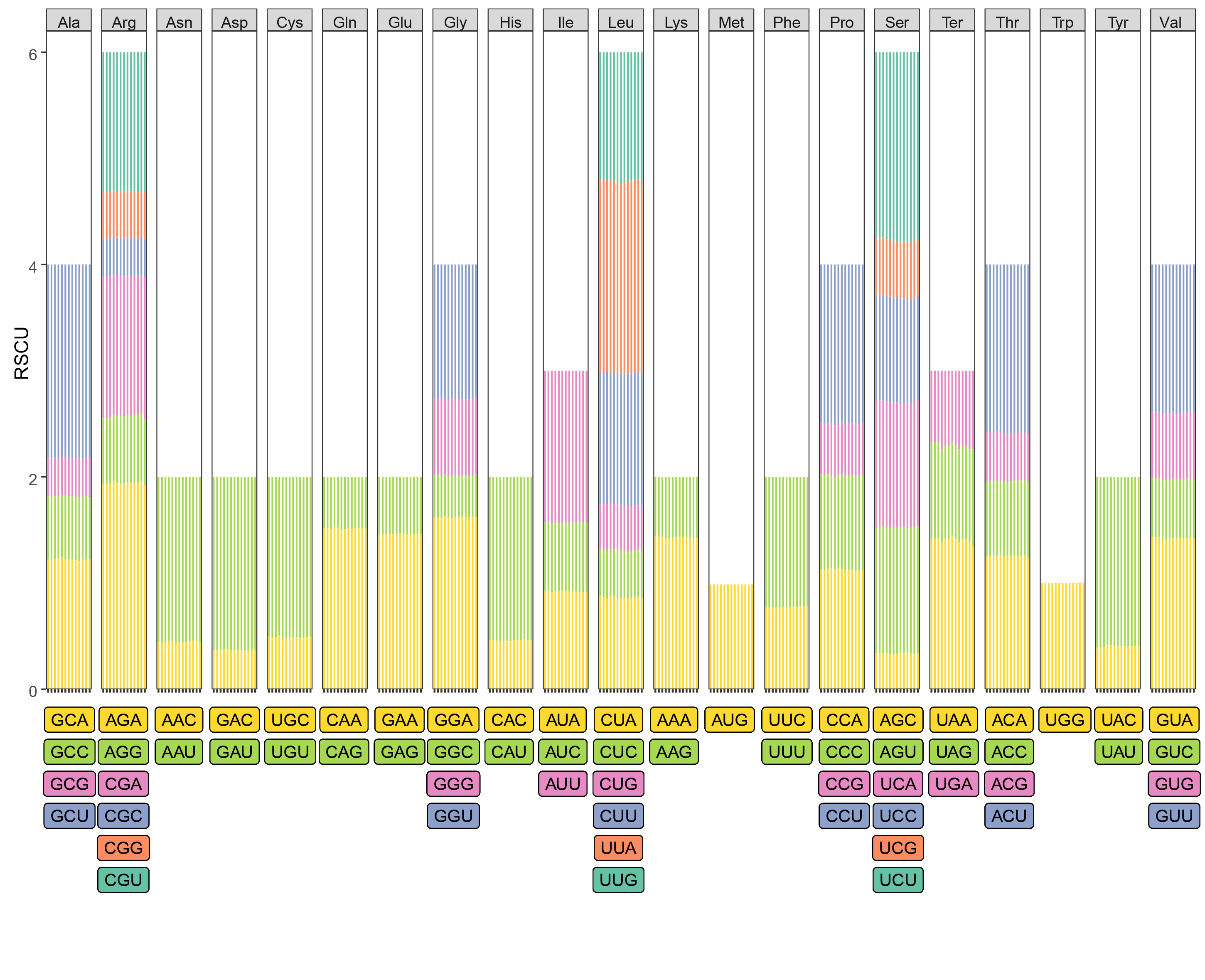

Supplement: Supplementary file 2 — Additional file 2: Figure S2. Codon usage frequency based on relative synonymous codon usage (RSCU) values in the thirteen Pholidota cp genomes. [file 12870_2023_4233_MOESM2_ESM.jpg]
